# Identification of initial severity determinants to predict arthritis after chikungunya infection in a cohort of French gendarmes

**DOI:** 10.1186/1471-2474-15-249

**Published:** 2014-07-24

**Authors:** Hafiz Muhammad Yaseen, Fabrice Simon, Xavier Deparis, Catherine Marimoutou

**Affiliations:** 1 Centre d’Epidémiologie et Santé Publique des Armées (CESPA), BP 40026, Marseille 13568 cedex 02, France; 2 UMR 912-SESSTIM, Aix-Marseille University, Marseille 13006, France; 3 Service de Pathologies infectieuses et tropicales, Hôpital d’instruction des Armées Laveran, CS 50004, Marseille 13384 cedex 13, France

**Keywords:** Chikungunya, Arthritis, Arthralgia, Recovery, Multiple correspondence analysis, Severity score

## Abstract

**Background:**

The objective was to identify severity characteristics of initial chikungunya infection (CHIKV) stages associated with post-CHIKV arthritis and arthralgia.

**Methods:**

French gendarmes exposed to the 2005–2006 CHIKV epidemic in Reunion Island who completed the 2006 (self-reporting acute and early chronic [median: 6 months] symptoms) and 2008 (Endpoint [median: 30 months]: self-perceived recovery and rheumatic disorders (RDs)) surveys were included. Multinomial logistic regression and multiple correspondence analysis (MCA) were used. Arthralgia was defined by joint pain and/or stiffness and arthritis by joint swelling in addition to pain and/or stiffness.

**Results:**

In 2008, 124 (31.3%)/403 participants (101 CHIKV+/302 CHIKV-) reported arthralgia and 57 (14.1%) arthritis. The multivariate model kept CHIKV infection, comorbidity and acute stage depressed mood as independent prognostic factors for both arthralgia and arthritis, but found early chronic stage RD as the main determinant of the same RD two years later.

The MCA performed with the 85 CHIKV + patients who answered the question on self-perceived recovery enabled the calculation of severity scores based on initial symptoms that were strongly associated with persistent arthritis and, to a lesser extent, to arthralgia in bivariate analyses. The MCA graph clearly distinguished arthritis as the only RD associated with early severity indicators represented by sick leave, joint swelling and depressed mood during the acute stage, and early chronification of arthritis and depressed mood.

**Conclusion:**

Initial CHIKV severity predicted recovery, with higher severity associated with arthritis and lower severity with arthralgia. More interestingly, specific markers of post-CHIKV arthritis, which can easily be used by clinicians for case management, were identified.

## Background

Chikungunya infection (CHIKV) is a self-limiting, two-stage disease caused by an *Aedes* mosquito-transmitted virus. The CHIKV acute stage typically consisting of fever, rash and multiple joint disorders is frequently followed by a chronic stage, mainly characterized by peripheral joint disorders [1-9], which it is estimated can persist up to five years [10]. Different studies have investigated prognostic factors of CHIKV non-recovery, generally assimilating non-recovery with the persistence of rheumatic disorders, but with non-standardized definitions of either “recovery” or “post-CHIKV rheumatic disorders” [3,11,12]. Moreover, if a severe acute stage of CHIKV was reported to be associated with persistent rheumatic disorders, no standard definition of severity was used [6,8,13]. No prognostic study distinguished between arthralgia and arthritis, although this distinction makes sense in terms of management. While arthralgia is the most common post-CHIKV rheumatic disorder (RD), arthritis is much more incapacitating and can be the first step towards destructive inflammatory rheumatism [14,15]. Thus, the identification of specific prognostic factors for post-CHIKV arthritis should be of interest as an aid to therapeutic decision-making to control the inflammation process early enough before any joint destruction occurs, as has recently been proposed by some rheumatologists [15,16].

In a previous study, we observed that, in a cohort of French gendarmes (members of a French military force responsible for carrying out police duties among civilian populations), CHIKV-infected patients (CHIKV+) presented a higher frequency and intensity of joint disorders and an impaired quality of life 30 months after infection, whether they self-considered themselves recovered or not, than non-infected (CHIKV-) subjects [2]. The objective of the present study was to identify specific prognostic factors of persistent arthritis in the same cohort, focusing on the symptoms of infection during the initial stages of the disease. In order to take into account the patients’ perception of the disease, the association of these factors with self-perceived recovery was also studied.

## Methods

### Ethical aspect

This study is based on data collected retrospectively at two different times among the same population of gendarmes. A major outbreak of CHIKV in Reunion Island ended in June 2006. In that context, it was decided that a study on the prevalence and consequences of infection among gendarmes would be conducted, because CHIKV was considered to be a possible work-related disease. All the gendarmes on duty in Reunion Island were offered a serological test for CHIKV and self-report questionnaires by their military physician. Testing was considered to be part of routine care, but patients were informed about the study by a written notice attached to the self-questionnaire and the military physician received their oral consent before inclusion. Only those who accepted the blood test and questionnaire were included. In 2008, follow-up questionnaires were offered to each participant of the 2006 study. Questionnaires were sent out by mail with written information on the study’s aim, which explained that it was an anonymous epidemiological study and instructed patients who agreed to participate, to send back the completed questionnaire using the pre-identified attached envelope. The two thirds of the patients who returned the questionnaires with self-reported, hand-written data were thus considered to have consented to participate in the study. Both the studies and their consent procedures were approved by the Clinical Research Committee of the Central Directorate of the Army Health Service (DCSSA). Data were analyzed anonymously.

### Study population and definitions

The study population was made up of the French gendarmes who completed both the 2006 survey (n = 662) [1] and the 2008 survey (n = 404) [2], minus one subject excluded for redundant data. The 259 non-respondents in 2008 did not differ from the 403 included in terms of proportion of CHIKV-infected patients, sex and age.

In accordance with previous studies performed on the same cohort [1,2], and due to the fact that serology results were highly concordant with self declaration; subjects were considered CHIKV + (n = 101) or CHIKV- (n = 302) according to their self declaration.

In both the 2006 and 2008 studies, participants were asked to mention which joints presented pain, stiffness or swelling at different times. To distinguish between arthralgia and arthritis, rheumatic disorders were classified in three categories: 1) no rheumatic disorders, if no pain, stiffness or swelling was reported, 2) arthralgia, if only joint pain and/or stiffness was reported, 3) arthritis, if joint swelling was reported in addition to pain and/or stiffness, regardless of the number of joints involved.

Data on initial CHIKV stages were those collected in the 2006 survey, which concerned acute stage symptoms and those persisting at the time of the survey (i.e. a median lapse of six months after CHIKV infection), the latter being called symptoms of the “early chronic stage” or “early chronic symptoms”. Clinical symptoms of the acute stage (fever, rash, joint pain, joint swelling, muscle pain, headache) were taken into account as dichotomous variables coded “yes” for present and “no” for absent. As fever was common, the level of fever (>39° or ≤39° according to the median), and its duration (>or ≤ 2 days [median duration]) were added. Asthenia was coded “yes” if it was self-evaluated as “quite significant”, “significant”, “very significant” or “totally disabling” on the 6-item Likert scale, and “no” if evaluated as “absent” or “minor”, and mood was coded “depressed” if it was self-evaluated as “affected” or “totally depressed” on the 4-item Likert scale, and “not depressed” if it was evaluated as “normal or weak but confident”. The notion of sick leave during the acute stage was added using two variables: sick leave (yes/no) and duration of sick leave (≤4 days or >4 days; [4 days = median duration]). Three items for the early chronic stage were kept: joint disorders (categorized as no rheumatic disorders/arthralgia/arthritis); asthenia (yes, no) and depressed mood (yes, no), using the same cut-off as that used on the Likert scales.

As age and rheumatic comorbidity have been associated with recovery in different studies [3,11,12], we also integrated in the models age (coded: ≤ 40 and >40 years, according to the median age of the participants in 2006), and a “comorbidity” variable coded “yes” if the patient had reported a history of “traumas in tendons, joints or bones” or “osteo-articular diseases”.

Finally, self-perceived recovery was defined by the answer “yes” or “no” to the 2008 survey question: “Do you consider yourself recovered?”.

### Statistical analysis

To identify the prognostic factors associated with persistent arthralgia or arthritis 30 months after infection (median duration of follow-up in 2008), we first analyzed the entire 2008 study sample using a multinomial logistic regression with “no rheumatic disorders” as the reference category. To implement the regression model, all the predictive variables were first assessed individually using univariate regression models. Then, variables with a significant p-value < 0.20 were included in the multivariate regression model, and a backward stepwise procedure was used to keep as predictors the variables with *p*-value <0.05. These regressions were performed using STATA software, version 9.

The CHIKV acute stage symptoms were largely shared by CHIKV + patients and absent in CHIKV- patients. This led to interactions and colinearity in the multivariate regression model. Identifying the independent predictive role of CHIKV infection in RDs was of epidemiological interest and keeping CHIKV- subjects in the study increased the power of the study, but with regard to the identification of CHIKV initial symptoms of severity that could help manage CHIKV + patients, keeping CHIKV- patients in analyses was useless. Thus, our second step was to focus on CHIKV + patients, and in order to avoid interaction and colinearity problems, a multiple correspondence analysis (MCA) was used. With the help of MCA, we sought to estimate predictors of both rheumatic disorders (separately for arthralgia and arthritis) and self-estimated recovery, but also to find a standardized definition of severity of early CHIKV infection which would be easy to identify and reproduce by clinicians. For these analyses, XL-Stat 9 and R 2.15.1 softwares were used.

The MCA displays the individuals and variable categories as labeled points in a multiple dimensional space. It decomposes all the information represented by variables and individuals into multiple factorial axes, such that each successive factorial axis captures a part of the total information in decreasing order [17]. Thus, the first two factorial axes capture the maximum amount of information available in the data. With the help of these two factorial axes, the graphic display of variable categories can reveal the structural relation between variables. In the graph, the more distant a variable category is from the origin, the higher its contribution is (i.e. the contribution of categories near the origin is small), but outliers situated too far from the origin are no longer informative. In the study, sex categories were thus excluded as there were only 3 females, which led to a distortion of the MCA graph (the category “women” representing an outlier that was too far from the origin). Most interestingly, the interpretation of data associations can easily be read on the MCA graph: the closer the variable categories are on the graph, the stronger they are associated. Here, the categories of self-perceived recovery at 30 months and rheumatic disorders were superimposed on the MCA graph to observe which symptoms were closer to (i.e. were associated with) self-perceived recovery, arthralgia and arthritis. The strength of these associations was tested in binary analyses using the Chi^2^ test.

One property of MCA is the possibility of providing a severity scale from 0 (corresponding to the theoretically least severe case) to 100 (corresponding to the theoretically most severe case) for each factorial axis [18]. The score is calculated using the following formula: [1-(X-Xmin)/(X-Xmax)]*100, where X = coordinate of an individual on a factorial axis F, Xmin = minimum coordinate on F, Xmax = maximum coordinate on F.

This formula was used to calculate 3 severity scores based on the MCA coordinates of variables on the first factorial axis, which was the most informative. The first score was calculated using both acute and early chronic stage symptoms, the second using only the acute symptoms and the third using only the early chronic symptoms. Associations between these scores and self-perceived recovery, arthritis and arthralgia at 30 months were also tested using Kruskal-Wallis tests.

## Results

### Multinomial regression analysis results

In 2008, 124 (31.3%) of the 403 participants reported arthralgia and 57 (14.1%) arthritis. It was more likely that they were CHIKV + than gendarmes with no RDs: 6 times more likely when reporting arthralgia and 11 times more when reporting arthritis. In addition, in the univariate analysis, all symptoms of initial CHIKV stages were significantly associated with persistent rheumatic disorders at 30 months, especially the rheumatic disorders of the early chronic stage. Arthralgia during the early chronic stage was 14 times more frequent in patients presenting arthralgia or arthritis in 2008 than in those with “no rheumatic disorders”. Arthritis at six months was 10 times more frequent in patients complaining of arthritis than in those complaining of arthralgia in 2008, and 40 times more frequent when comparing the 2008 arthritis group to the “no RDs” group. Moreover, patients with rheumatic disorders in 2008 also declared rheumatic comorbidities 1.3 times more frequently.

The final multivariate model excluding the early chronic stage symptoms kept the following as independent prognostic factors: CHIKV infection, comorbidity and acute stage depressed mood for both persistent arthralgia and arthritis. When the early chronic symptoms were added to the model, the role of the acute stage symptoms disappeared in favor of a strong association between the rheumatic disorders present during the early chronic stage and the presence of the same RDs two years later (i.e. in 2008; Table 1).

**Table 1:** Prognostic factors associated with rheumatic symptoms in 2008, cohort of French Gendarmes (2006–2008), n = 403 (Multinomial Logistic Regression Analysis)

	**Multivariate analysis (without early chronic symptoms)**		**Multivariate analysis (with early chronic symptoms)**	
	**Reference: no rheumatic disorders OR (95% CI)**		**Reference: no rheumatic disorders OR (95% CI)**	
	**Arthralgia**	**Arthritis**	**Arthralgia**	**Arthritis**
CHIKV Infection	4.67 (2.32-9.41)ǂ	26.75 (11.15-64.17)ǂ	2.58 (1.05-6.38)*	10.56 (3.42-32.63)ǂ
Comorbidity^a^	2.00 (1.25-3.20)ǂ	2.47 (1.19-5.14)*	2.03 (1.26-3.27)ǂ	2.27 (1.06-4.87)*
*Symptoms at acute stage*				
Depressed mood^b^	8.90 (1.06-74.95)*	13.92 (1.66-116.88)*	-	-
*Symptoms at early chronic stage (median: 6 months after infection)*				
Arthralgia^c^	-	-	8.64 (1.95-38.17)ǂ	6.65 (1.33-33.30)*
Arthritis^d^	-	-	2.93 (0.47-18.18)	19.38 (3.36-111.82)ǂ

*p < 0.05; ǂp < 0.005; OR: odds ratio; CI: confidence interval.

^a^History of at least one of the following pathologies: sprain or dislocation of joints or bones and joint diseases, before CHIKV infection.

^b^Self-assessed mood coded as “depressed” if checked affected or totally depressed.

^c^Arthralgia = joint pain and/or stiffness.

^d^Arthritis = swelling in addition to joint pain and stiffness.

### MCA analysis results

**Figure 1:**
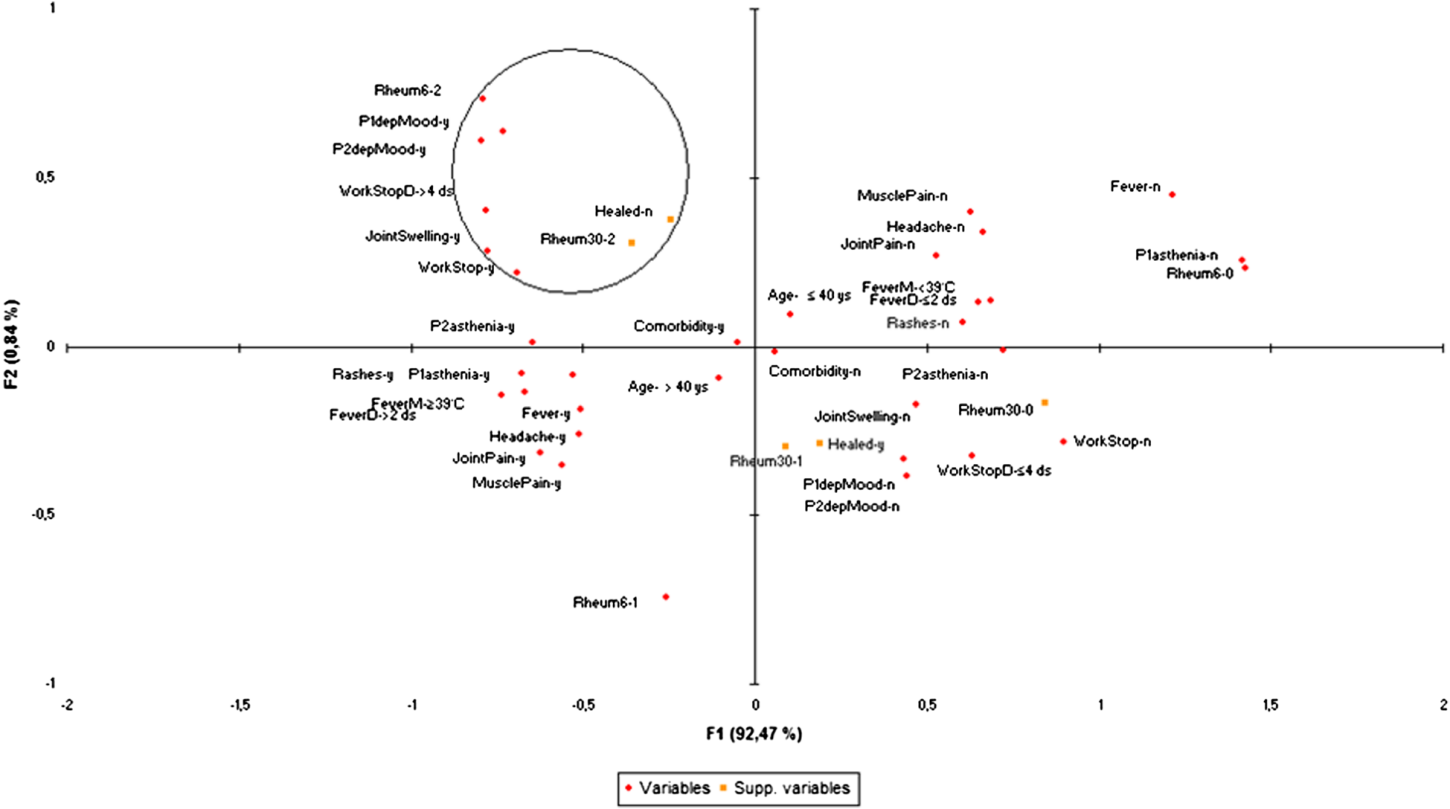
**Multiple Correspondence Analysis graph with CHIKV acute and early chronic stage symptoms, Cohort of French CHIKV+ Gendarmes (2006–2008, N = 85).** Legend: Rheum6-0, Rheum6-1, Rheum6-2 = respectively No rheumatic disorders, arthralgia, arthritis at 6 months; P1 = acute stage; P2 = early chronic stages; D = duration; M = maximum; y = yes; n = no; Workstop: sick leave. depMood: depressed mood; Supp. variables=supplementary variables=recovery (y-healed/n-healed) and rheumatic symptoms at 30 months (Rheum30-0, Rheum30-1, Rheum30-2 = respectively No rheumatic disorders, arthralgia, arthritis at 30 months).

For this analysis, the 16/101 CHIKV + non-responders to the question on recovery were excluded. The study sample was therefore 85 patients, 48 (56%) declaring themselves recovered in 2008 and 37 (44%) not recovered. Patients were 82/85 (96.5%) male and the median age was 40 years (range: 21–54 years) at disease onset. During the acute stage, 60 patients (71%) reported fever, 42 (49%) fever >39°C and 41 (49%) fever duration >2 days; 40 (47%) reported rash, 39 (46%) joint pain, 32 (38%) joint swelling, 45 (53%) muscle pain, 48 (57%) headache, 48 (57%) sick leave due to CHIKV infection (38 [45%] for more than 4 days), 62 (73%) reported significant asthenia and 30 (35%) depressed mood. During the early chronic stage, 35 (42%) patients reported arthralgia, 29 (33%) arthritis, 45 (53%) significant asthenia and 32 (38%) depressed mood. In 2008, 13 (15%) patients reported no RDs, 33 (39%) reported arthralgia and 39 (46%) arthritis.The MCA results are presented in Figure 1. Factorial axis 1 captured 93.1% of the variability and clearly distinguished the presence of initial CHIKV symptoms on the left from absence on the right. Age, sex and comorbidity did not participate in severity scores as they were not significantly associated with axis 1, but only poorly associated with axis 2. It can be seen in Figure 1 that age and comorbidity categories are close to the origin of the axes.

The bivariate analysis testing the association between three severity scores calculated on axis 1 and rheumatic symptoms is summarized in Table 2 and in Table 3 for self-perceived recovery. The three scores were significantly associated with any persistent rheumatic disorders, but the median scores were higher and the association stronger with arthritis than with arthralgia (Table 2). Only the early chronic stage score was associated with self-perceived non-recovery (Table 3).According to the MCA graph (Figure 1), the following symptoms: sick leave, its duration >4 days, joint swelling and depressed mood during acute stage, in addition to early chronic arthritis and depressed mood, circled in the upper left corner of Figure 1, were closely associated with persistent arthritis at 30 months and self-perceived non-recovery at that time (categories close to each other in the same circle). Arthralgia at 30 months was projected in the right lower quarter of the graph, i.e. on the side of “no or mild” symptoms of initial CHIKV infection.

**Table 2:** Distribution of initial severity scores calculated with the first factorial axis of the multiple correspondence analysis according to rheumatic disorders reported by CHIKV + patients in 2008 (N = 85), cohort of French Gendarmes (2006–2008)

	**No rheumatic disorders**^**d**^	**Arthralgia**^**d**^	p*	**Arthritis**^**d**^	p*
	**N = 13 (15.3%)**	**N = 33 (38.8%)**		**N = 39 (45.9%)**	
Median overall severity score (IQ)	0 (0–39.7)	60.8 (22.3-79.6)	0.024	73.2 (60.9-82.3)	0.001
Median acute stage severity score (IQ)	0 (0–43.3)	60.0 (24.9-78.8)	0.042	75.4 (56.5-82.0)	0.002
Median early chronic stage severity score (IQ)	0 (0–61.7)	61.7 (31.1-89.1)	0.025	72.6 (50.3-100.0)	0.001

IQ: interquartile 25%-75%.

*Kruskal-Wallis test comparing the distribution of severity scores according to rheumatic symptoms, considering “No rheumatic disorders” as reference.

^d^Arthralgia = joint pain and/or stiffness. Arthritis = swelling in addition to joint pain and stiffness.

**Table 3:** Distribution of initial severity scores calculated with the first factorial axis of the multiple correspondence analysis according to self-perceived recovery status of CHIKV + patients in 2008 (N = 85), cohort of French Gendarmes (2006–2008)

	**Recovered**	**Non-recovered**	p*
	**N = 48 (56.47%)**	**N = 37 (43.5%)**	
Median overall severity score (IQ)	55.6 (0–78.7)	70.7 (49.1-79.6)	0.098
Median acute stage severity score (IQ)	58.0 (0–78.5)	67.0 (44.5-79.0)	0.202
Median early chronic stage severity score (IQ)	58.5 (0–72.6)	72.6 (58.5-100)	0.006

IQ: interquartile 25%-75%.

*Kruskal-Wallis test comparing the distribution of severity scores according to recovery.

The strong association of the circled symptoms with arthritis at 30 months was confirmed by bivariate Chi^2^ analysis (Table 4). The associations were weaker with self-perceived non-recovery, and not confirmed for acute stage sick leave >4 days and acute joint swelling (Table 5).

**Table 4:** **Bivariate analysis (Chi**^**2 **^**test) testing the association between the initial symptoms of Chikungunya infection associated with long-term arthritis on the MCA Graph and rheumatic disorders declared at 30 months by CHIKV + Patients (N = 85), Cohort of French Gendarmes (2006–2008)**

	**No rheumatic disorders**	**Arthralgia**^**c**^	P*	**Arthritis**^**c**^	P**
	**N = 13 (15.3%)**	**N = 33 (38.8%)**		**N = 39 (45.9%)**	
Comorbidity^a^	4 (30.8)	16 (48.5)	0.447	25 (64.1)	0.076
*Symptoms at acute stage*	-	-		-	-
Sick leave (yes)	3 (23.1)	17 (51.5)	0.155	28 (71.8)	0.006
Duration of absence from work >4 days	2 (15.4)	13 (39.4)	0.224	23 (59.0)	0.016
Joint swelling (yes)	2 (15.4)	9 (27.3)	0.640	21 (53.9)	0.036
Depressed mood^b^	-	11 (33.3)	0.045	19 (48.7)	0.005
*Symptoms at early chronic stage (median: 6 months after infection)*					
Rheumatic symptoms^c^					
Arthralgia	3 (23.1)	21 (63.6)	0.025	11 (28.2)	0.122
Arthritis	2 (15.4)	4 (12.1)	0.827	22 (56.4)	0.004
Depressed mood^b^	-	11 (33.3)	0.045	21 (53.8)	0.002

^*χ2^ test of comparison between No rheumatic disorders and Arthralgia.

**^χ2^ test of comparison between No rheumatic disorders and Arthritis.

^a^History of at least one of the following pathologies: sprain or dislocation of joints or bones and joint diseases, before CHIK infection.

^b^Self-assessed mood coded as “depressed” if checked affected or totally depressed.

^c^Arthralgia = joint pain and/or stiffness. Arthritis = swelling in addition to joint pain and stiffness.

**Table 5:** **Bivariate Analysis (Chi**^**2 **^**test) testing the association between the initial symptoms of Chikungunya infection associated with non-recovery on the MCA graph and self-perceived recovery status at 30 months by CHIKV + patients (N = 85), Cohort of French Gendarmes (2006–2008)**

	**Recovered**	**Non-recovered**	p*
	**N = 48 (56.47%)**	**N = 37 (43.5%)**	
Comorbidity^a^	24 (50.0)	21 (56.8)	0.689
*Symptoms at acute stage*	-	-	-
Sick leave (yes)	22 (45.8)	26 (70.3)	0.042
Duration of sick leave >4 days	17 (35.4)	21 (56.8)	0.082
Joint swelling	17 (35.4)	15 (40.5)	0.797
Depressed mood^b^	11 (22.9)	19 (51.4)	0.012
*Symptoms at early chronic stage (median: 6 months after infection)*			
Rheumatic symptoms^c^			
Arthralgia	23 (47.9)	12 (32.4)	1.000
Arthritis	10 (20.8)	18 (48.6)	0.046
Depressed mood^c^	12 (25.0)	20 (54.1)	0.012

^*χ2^ test of comparison of distribution of each characteristics between the two groups.

^a^History of at least one of the following pathologies: sprain or dislocation of joints or bones and joint diseases, before CHIK infection.

^b^Self-assessed mood coded as “depressed” if checked affected or totally depressed.

^c^Arthralgia = joint pain and/or stiffness. Arthritis = swelling in addition to joint pain and stiffness.

## Discussion

Both analyses underlined the determinative role of acute depressed mood and early chronification of arthritis in the risk of persistent arthritis at 30 months. However, when CHIKV- patients were taken into account (multiple regression model), the main determinants of long-term rheumatic disorders whether inflammatory or not were CHIKV infection and underlying rheumatic comorbidities. The MCA restricted to CHIKV + patients enabled us to demonstrate that CHIKV initial symptoms were highly interrelated, and to calculate severity scores that were strongly associated with persistence of arthritis after 30 months. Moreover, it made it possible to isolate simple initial severity indicators easily identifiable by clinicians for patient management, such as long sick leave and joint swelling during the acute stage, in addition to chronification of depressed mood and arthritis at six months. Although long term arthralgia was linked to underlying diseases according to the first analysis, the MCA did not find it to be associated with CHIKV severity.

The association between severity of the acute CHIKV stage and persistent rheumatic disorders has already been described by some studies [6,8,13]. But the present study is the first to quantify the overall severity of the acute and early chronic stages of CHIKV infection in the form of severity scores, and thus to objectify the association between initial severity and long-term post-CHIKV rheumatic disorders and self-perceived recovery. These scores showed that severity of both acute and early chronic stages of CHIKV infection was associated with long-term rheumatic disorders. However, the association was much stronger and the severity scores higher for long-term arthritis than for arthralgia, suggesting that the more severe the initial CHIKV infection, the more likely it was that patients would develop long-term arthritis. At a mild level of severity, they would develop arthralgia (with a far lower risk of joint destruction), and at a lower level no rheumatic consequence. The location of 30-month arthralgia on the MCA graph confirmed this hypothesis.

In addition, our study is the first to distinguish common arthralgia from arthritis among post-CHIKV rheumatic disorders, which made it possible to identify specific prognostic factors of arthritis. In a recent study, Gérardin *et al.*[19] distinguished relapsing post-CHIKV rheumatic musculoskeletal pain (RMSP) from lingering RMSP based on the frequency of episodes of pain. They found that age and initial severity (defined by severe rheumatic involvement and level of CHIKV IgG titers during the acute phase) were predictive of both lingering and relapsing RMSP [19]. The distinction based on intensity of rheumatic disorders, arthritis being more severe than arthralgia, seems more relevant, since it made it possible to isolate specific predictors and has consequences for patient management, in particular for therapeutic decisions [15,16]. The study was based on self-reported symptoms, so the diagnosis of arthritis cannot be certain. However, after discussion with clinicians, swelling was considered specific enough to synovitis to warrant proposing this distinction. Moreover, the consistency of data between 2008 and 2006, as well as with incapacitation reported on the quality of life SF-36 questionnaire completed in 2008 (data not shown), reinforced the reliability of patients’ declarations.

The MCA graph enabled an easy visual identification of associations between certain symptoms and long-term arthritis or self-perceived non-recovery. Some of the symptoms identified, such as joint swelling during the acute stage, have already been reported as being associated with CHIKV recovery [11], but with a different definition of recovery. In our results, there was a clear continuum in the inflammatory symptoms, as swelling in the acute stage was also found in the early and late chronic stages of CHIKV. Depression was reported to be frequent after CHIKV infection [2,4]. In our study, depressed mood during both acute and early chronic stages were prognostic of both persistence of arthritis at 30 months and self-reported non-recovery. This link suggests that attention should be paid to this symptom, which may be indicative of either the severity of the disease or the patient’s anxiety concerning delay in recovery. However, a direct action of the CHIK virus on the brain should be explored.

The MCA found other indirect markers of severity associated with long-term arthritis, such as absence from work and its duration. This is consistent with the notion that the more severe the acute stage is, the greater the risk will be to develop arthritis. Last but not least, reporting arthritis during the early chronic stage was found to be associated with the long-term persistence of arthritis, while early arthralgia was not. This was also found with the multinomial logistic regression, which identified early chronic arthralgia as being associated with late persistent arthralgia and early arthritis with late arthritis. This fact is of major importance, because it supports the recommendation that early detection of persistent arthritis within the first 6 months should be carried out in order to initiate adequate treatment. Rheumatologists in Reunion Island and India have recommended that the treatment should be based on disease-modifying antirheumatic drugs, with methotrexate as the first-line therapy to control the inflammation process before joint destruction occurs [15,16].

In no analysis was age contributive, although it has been widely reported to be associated with CHIKV recovery [3,8,11]. This might be due to the specificity of our population, made up of young, healthy workers, none of whom were over 55 years of age.

Moreover, according to the MCA, the contribution of comorbidity was very small, although it was also frequently reported [3,8,11]. The frequency of previous musculoskeletal pain or traumatic history among patients suffering from post-CHIKV rheumatic disorders often led to the consideration that CHIKV may reveal or stimulate underlying rheumatic diseases. Our results on the entire cohort sample are in favor of this conclusion, as the association between rheumatic comorbidity and late rheumatic disorders existed for both infected and non-infected patients. It seemed more likely that the association could be explained by arthralgia, as it was the most frequent rheumatic disorder when considering CHIKV- subjects and comorbidity did not contribute to long-term arthritis according to the MCA.

The two different assessments of recovery (self-perceived or evaluated by persistent rheumatic disorders at 30 months) led to differences in the prognostic factors found to be associated with recovery. Self-perceived recovery was only associated with the severity score based on “early chronic stage” symptoms, suggesting that the severity of entry into the chronic stage is the main predictor of the perception of recovery. Since the question about recovery was asked 30 months after the acute infection, it seems logical that patients who did not enter the chronic stage (no or few symptoms reported after six months of infection) considered themselves recovered regardless of the severity of their acute stage. On the other hand, most of the subjects who declared themselves recovered did report rheumatic disorders (35% reported arthralgia and 37% arthritis, data not shown). They may either have considered that their symptoms were not linked to CHIKV or considered that they were a bearable after-effect. It is therefore up to physicians to estimate the severity of symptoms and their possible consequences on joint integrity in order to decide on future management. The existence of severity characteristics during the previous stages of CHIKV disease should be an additional aid to therapeutic decision-making.

## Conclusions

The severity of initial stages of CHIKV infection negatively influences long-term recovery and in particular increases the risk of long-term arthritis. However, the severity scores provided by the MCA cannot be directly used by physicians to predict how the condition of their individual patients will progress, as they cannot be calculated for a single subject. For the management of an individual patient’s condition, asking about the presence of joint swelling, mood depression and lengthy absence from work during the acute stage may alert physicians to the risk of arthritis in the medium- and long-term. The early chronification of arthritis should lead to further investigations, so that the physician can make rapid decisions about specific therapies to avoid long-term persistence and risk of joint destruction.

## Competing interests

The authors declared no competing interests.

## Authors’ contributions

HMY performed the statistical analysis and wrote the first draft of the manuscript. FS designed and coordinated the cohort study and helped to draft the manuscript. XD participated in the present study design and supervision of the statistical analysis and helped draft the manuscript. CM participated in the design and coordination of the cohort study, in the design of the present study and supervision of the analyses and was in charge of the final draft of the manuscript. All authors read and approved the final manuscript.

## Pre-publication history

The pre-publication history for this paper can be accessed here:

http://www.biomedcentral.com/1471-2474/15/249/prepub

## References

[B1] Queyriaux B Simon F Grandadam M Michel R Tolou H Boutin JP Clinical burden of chikungunya virus infection Lancet Infect Dis 2008 8 1 2 3 10.1016/S1473-3099(07)70294-3 18156079

[B2] Marimoutou C Vivier E Oliver M Boutin JP Simon F Long-lasting overmorbidity and impaired quality of life 30 months after chikungunya infection in La Reunion Island: comparative cohort of infected and uninfected French military policemen Medicine 2012 91 212 219 10.1097/MD.0b013e318260b604 22732952

[B3] Sissoko D Malvy D Ezzedine K Renault P Moscetti F Ledrans M Pierre V Post-epidemic Chikungunya disease on Reunion Island: course of rheumatic manifestations and associated factors over a 15-month period PLoS Negl Trop Dis 2009 3 3 e389 10.1371/journal.pntd.0000389 19274071 PMC2647734

[B4] Soumahoro MK Gerardin P Boelle PY Perrau J Fianu A Pouchot J Malvy D Flahault A Favier F Hanslik T Impact of Chikungunya virus infection on health status and quality of life: a retrospective cohort study PLoS One 2009 4 11 e7800 10.1371/journal.pone.0007800 19911058 PMC2771894

[B5] Manimunda SP Vijayachari P Uppoor R Sugunan AP Singh SS Rai SK Sudeep AB Muruganandam N Chaitanya IK Guruprasad DR Clinical progression of chikungunya fever during acute and chronic arthritic stages and the changes in joint morphology as revealed by imaging Trans R Soc Trop Med Hyg 2010 104 6 392 399 10.1016/j.trstmh.2010.01.011 20171708

[B6] Borgherini G Poubeau P Jossaume A Gouix A Cotte L Michault A Arvin-Berod C Paganin F Persistent arthralgia associated with chikungunya virus: a study of 88 adult patients on Reunion Island Clin Infect Dis 2008 47 4 469 475 10.1086/590003 18611153

[B7] Gérardin P Fianu A Malvy D Mussard C Boussaïd K Rollot O Michault A Gaüzere BA Bréart G Favier F Perceived morbidity and community burden after a Chikungunya outbreak: the TELECHIK survey, a population-based cohort study BMC Med 2011 9 5 10.1186/1741-7015-9-5 21235760 PMC3029216

[B8] Larrieu S Pouderouxb N Pistoneb T Filleul L Receveurb MC Sissoko D Ezzedineb K Malvy D Factors associated with persistence of arthralgia among chikungunya virus-infected travellers: Report of 42 French cases J Clin Virol 2010 47 85 88 10.1016/j.jcv.2009.11.014 20004145

[B9] Simon F Javelle E Oliver M Leparc-Goffart I Marimoutou C Chikungunya Virus Infection Curr Infect Dis Rep 2011 13 218 228 10.1007/s11908-011-0180-1 21465340 PMC3085104

[B10] Yaseen HM Simon F Deparis X Marimoutou C Estimation of lasting impact of a Chikungunya outbreak in reunion island Epidemiol 2012 S2 003 doi:104172/2161-1165S2-003

[B11] Couturier E Guillemin F Mura M Leon L Virion JM Letort MJ De Valk H Simon F Vaillant V Impaired quality of life after chikungunya virus infection: a 2-year follow-up study Rheumatology (Oxford) 2012 51 1315 1322 10.1093/rheumatology/kes015 22427407

[B12] Mohd Zim MA Sam I-C Syed Omar SF Chan YF AbuBakar S Kamarulzaman A Chikungunya infection in Malaysia: comparison with dengue infection in adults and predictors of persistent arthralgia J Clin Virol 2013 56 141 145 10.1016/j.jcv.2012.10.019 23201456

[B13] Chow A Her Z Ong EK Chen JM Dimatatac F Kwek DJ Barkham T Yang H Renia L Leo YS Ng LF Persistent arthralgia induced by Chikungunya virus infection is associated with interleukin-6 and granulocyte macrophage colony-stimulating factor J Infect Dis 2011 203 2 149 157 10.1093/infdis/jiq042 21288813 PMC3071069

[B14] Bouquillard E Combe B A report of 21 cases of rheumatoid arthritis following Chikungunya fever. A mean follow-up of two years Joint Bone Spine 2009 76 6 654 657 10.1016/j.jbspin.2009.08.005 19945329

[B15] Mathew AJ Goyal V George E Thekkemuriyil DV Jayakumar B Chopra A Rheumatic-musculoskeletal pain and disorders in a naive group of individuals 15 months following a Chikungunya viral epidemic in south India: a population based observational study Int J Clin Pract 2011 65 12 1306 1312 10.1111/j.1742-1241.2011.02792.x 22093538

[B16] Ribera A Degasne I Jaffar Bandjee MC Gasque P Chronic rheumatic manifestations following chikungunya virus infection: clinical description and therapeutic considerations Med Trop 2012 72 83 85 22693935

[B17] Greenacre M Correspondance Analysis In Practice 2007 Secondth Boca Raton, Florida, USA: Chapman & Hall/CRC Taylor & Francis Group 275

[B18] Murgue B Deparis X Chungue E Cassar O Roche C Dengue: an evaluation of dengue severity in French Polynesia based on an analysis of 403 laboratory-confirmed cases Trop Med Int Health 1999 4 11 765 773 10.1046/j.1365-3156.1999.00478.x 10588771

[B19] Gérardin P Fianu A Mussard C Boussaid K Rollot O Grivard P Kassab S Bouquillard E Borgherini G Gauzere BA Malvy D Breart G Favier F Predictors of chikungunya rheumatism: a prognostic survey ancillary to the TELECHIK cohort study Arthritis Res Ther 2013 15 R9 doi:10.1186/ar4137 10.1186/ar4137 23302155 PMC3672753

